# Microbial electrosynthesis of methane and acetate—comparison of pure and mixed cultures

**DOI:** 10.1007/s00253-022-12031-9

**Published:** 2022-06-28

**Authors:** Jan-Niklas Hengsbach, Björn Sabel-Becker, Roland Ulber, Dirk Holtmann

**Affiliations:** 1 Department of Mechanical and Process Engineering, Institute of Bioprocess Engineering, Technical University Kaiserslautern, 67663 Kaiserslautern, Germany; 2 Department of Life Science Engineering, Institute of Bioprocess Engineering and Pharmaceutical Technology, Technische Hochschule Mittelhessen, 35390 Giessen, Germany

**Keywords:** Electrobiotechnology, Microbial electrosynthesis, Pure culture, Mixed culture, Acetate, Methane

## Abstract

**Abstract:**

The electrochemical process of microbial electrosynthesis (MES) is used to drive the metabolism of electroactive microorganisms for the production of valuable chemicals and fuels. MES combines the advantages of electrochemistry, engineering, and microbiology and offers alternative production processes based on renewable raw materials and regenerative energies. In addition to the reactor concept and electrode design, the biocatalysts used have a significant influence on the performance of MES. Thus, pure and mixed cultures can be used as biocatalysts. By using mixed cultures, interactions between organisms, such as the direct interspecies electron transfer (DIET) or syntrophic interactions, influence the performance in terms of productivity and the product range of MES. This review focuses on the comparison of pure and mixed cultures in microbial electrosynthesis. The performance indicators, such as productivities and coulombic efficiencies (CEs), for both procedural methods are discussed. Typical products in MES are methane and acetate, therefore these processes are the focus of this review. In general, most studies used mixed cultures as biocatalyst, as more advanced performance of mixed cultures has been seen for both products. When comparing pure and mixed cultures in equivalent experimental setups a 3-fold higher methane and a nearly 2-fold higher acetate production rate can be achieved in mixed cultures. However, studies of pure culture MES for methane production have shown some improvement through reactor optimization and operational mode reaching similar performance indicators as mixed culture MES. Overall, the review gives an overview of the advantages and disadvantages of using pure or mixed cultures in MES.

**Key points:**

• *Undefined mixed cultures dominate as inoculums for the MES of methane and acetate, which comprise a high potential of improvement*

• *Under similar conditions, mixed cultures outperform pure cultures in MES*

• *Understanding the role of single species in mixed culture MES is essential for future industrial applications*

## Introduction

Decarbonization and electrification of the chemical industry are current tasks of the scientific community. To achieve the full potential in terms of decarbonization and electrification, CO_2_ should be the initial substrate and the applied electricity should be generated from renewable sources (e.g., solar or wind). In order to fulfil these requirements, microbial electrosynthesis (MES) theoretically represent an ideal technology platform to achieve this objective by combining microbial and electrochemical reactions. A common and widely applicable definition of these processes is “MES is the execution of microbially catalyzed electrochemical reactions to transform a substance into a desired product” (Schröder et al. 2015). The main advantages of MES compared to other power-to-X technologies are the mild reaction conditions (temperature and pressure), the high stability of the biocatalysts as well as their ability to self-regenerate and to synthesize complex molecules.

By combining an efficient CO_2_ utilization technology with renewable energy, the different United Nations Sustainable Development Goals (SDGs) could be fulfilled (Stöckl et al. 2022). MES can particularly contribute to achieve SDG 9 (Build resilient infrastructure, promote inclusive and sustainable industrialization and foster innovation), SDG 12 (Ensure sustainable consumption and production patterns), and SDG 13 (Take urgent action to combat climate change and its impacts). The feasibility of MES has been demonstrated several times in recent years (e.g., Nevin et al. (2010), Marshall et al. (2012), Batlle-Vilanova et al. (2017), Krieg et al. (2018), Vassilev et al. (2018)). However, to achieve the overall objectives, adequate process performances must be realized. The most important performance indicators in microbial electrosynthesis are the space-time yields, the current as well as the energy efficiencies, and the production rates based on the cathode surface area. Furthermore, additional parameters such as the conversion rate of the substrate, the cell voltage and in particular the investment and operating costs must always be considered.

The overall performance of MES is influenced by many different factors, e.g., the metabolic capacity of the organism, the type of electron transfer between the electrode and the organisms (Sydow et al. 2014; Stöckl et al. 2022), the type and area of the electrode, as well as the applied reactors (Krieg et al. 2014). In recent literature, examples of MES with both pure cultures and mixed cultures have been presented (Das et al. 2018; Gomez Vidales et al. 2019; Ragab et al. 2019; Mayer et al. 2019; Roy et al. 2021). The review aims to compare the performance indicators of these processes to identify the influence of these operational conditions. Typical products in MES, using CO_2_ as carbon source, are methane and acetate. Therefore, these processes are the focus of this review.

### Acetogens and methanogens

Both the metabolism and the technical use of acetogens and methanogens have been summarized in many review articles (e.g., Jones et al. 1987; Thauer et al. 2008; Schiel-Bengelsdorf and Dürre 2012; Schuchmann and Müller 2014; Costa and Leigh 2014; Schuchmann and Müller 2016; Enzmann et al. 2018; Lyu et al. 2018). Here, the most important characteristics of these organisms are briefly described. For more detailed information, please refer to the corresponding reviews.

To define an acetogen, the feature of “acetogenesis” in acetogens must be clearly distinguished from the sole ability to produce acetate. Different organisms, such as enterobacteria or acetic acid bacteria, could produce acetate, but are not acetogens. Acetogens are bacteria that can produce acetyl-CoA (and from that, in most cases, acetate as the end product) from two molecules of CO_2_ and, thus, from inorganic carbon, catalyzed by the reactions of the Wood-Ljungdahl pathway. Therefore, acetogenic bacteria can be defined as a diverse group of strictly anaerobic bacteria, which utilize the Wood-Ljungdahl pathway for the CO/CO_2_ fixation via acetyl-CoA. Acetogens are facultative autotrophs that can grow by the oxidation of a large variety of organic substrates (e.g., hexoses, pentoses, alcohols) or by the oxidation of inorganic substrates, such as H_2_ or CO, which is usually coupled to the reduction of CO_2_ (Schuchmann and Müller 2014). The most characteristic feature of acetogens is their ability to produce acetate from H_2_ and CO_2_. As the synthesis of acetate from 2 mol of CO_2_, with H_2_ as the reductant, enables the growth of acetogens, this pathway must be coupled to a net adenosine triphosphate (ATP) formation. Indeed, the Wood-Ljungdahl pathway is the only pathway for a CO_2_ fixation that is coupled to energy conservation (Schuchmann and Müller 2014). This reaction provides only limited energy for the cell metabolism, e.g., 0.3 mol ATP are generated per mol produced acetate by *Acetobacterium woodii* growing on CO_2_ and H_2_. Typical organisms that are counted among the acetogens belong to the genera *Clostridium*, *Acetobacterium*, and the thermophilic genera *Moorella* (Liew et al. 2016). Well-known model organisms of the acetogens are *Moorella thermoacetica*, *A. woodii*, *Sporumusa ovata*, and *Clostridium ljungdahlii*.

Methanogenesis is an anaerobic respiration that generates methane as the final product of metabolism (Sowers 2009; Lyu et al. 2018). The diverse archaeal group of methanogens is the only group of microorganisms on earth that produces significant amounts of methane (Enzmann et al. 2018). In general, methanogens are strict anaerobes. This group of organisms uses CO_2_ and H_2_ and/or small organic molecules, such as acetate, formate, and methylamine, and converts them into methane. In methanogenesis, the oxidized carbon compounds are used as terminal electron acceptors. Thus, methanogens are common in habitats that are poor in other electron acceptors, such as NO_3_^–^, Fe_3_^+^, and SO_4_^2–^ (Lyu et al. 2018). Therefore, this process occurs in anaerobic natural habitats (e.g., swamps, digestive systems of animals, oil fields) as well as in anaerobic technical systems (e.g., wastewater treatment and biogas plants). Methanogens can use three main types of substrates, namely CO_2_/CO, acetate, and/or methylated substrates. Based on these groups of converted substrates, methanogens are classified into three groups: hydrogenotrophic, acetoclastic, and methylotrophic methanogens. Most of the methanogens use CO_2_ as a carbon source, and H_2_ as an electron donor during hydrogenotrophic methanogenesis. Some methanogens can also use carbon monoxide (CO) for methanogenesis. The acetoclastic methanogens split acetate to form CH_4_ and CO_2_. Methylotrophic methanogenesis results from the demethylation of methanol and further compounds such as trimethylamine and dimethylamine. In general, the energy yield in methanogenesis is quite low (≤ 1 ATP per methane is generated) (Lyu et al. 2018). Methanogens show not only a wide diversity regarding their habitats but are also highly diverse in terms of morphology and growth conditions, such as temperature, pH, and osmolarity optima.

### Electron transfer and types of interspecies interactions

**Fig. 1:**
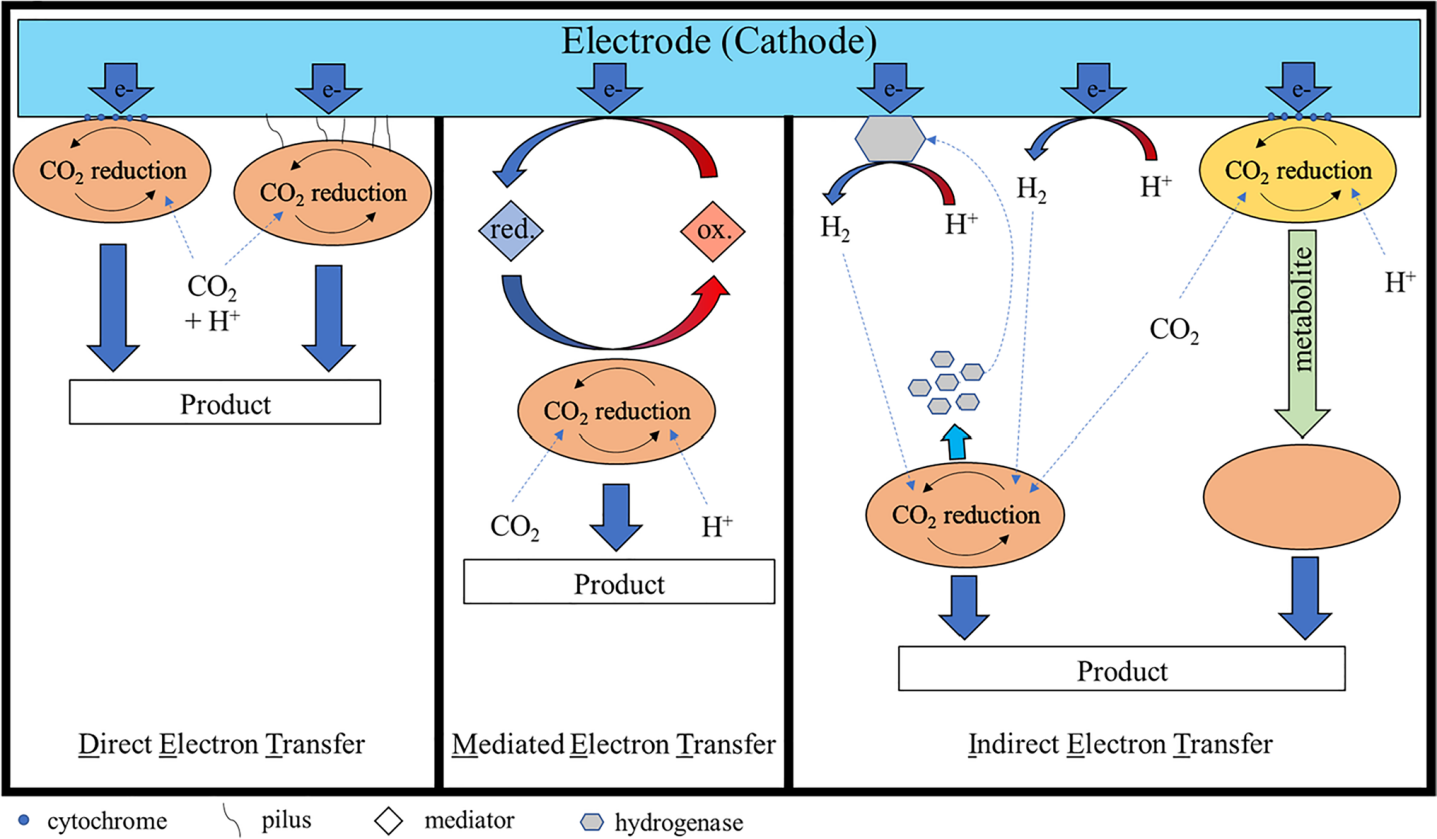
The different extracellular electron transfer mechanisms (EET) are illustrated showing the direct electron transfer (DET), the mediated electron transfer (MET), and the indirect electron transfer (IET). The physical contact in the DET can be accomplished through cytochrome (left), pili (right), and nanowire (not shown). The IET is possible through, e.g., proton (H+) reduction to hydrogen (H2) (uncatalyzed or catalyzed by hydrogenases) or a metabolite of another organism that functions as a substrate for the production strain

A main feature of electroactive bacteria is the ability to transfer electrons from an electrode to the microbial cell or vice versa instead of the natural redox partner (Sydow et al. 2014). For this, different extracellular electron transfer mechanisms can be employed (Fig. 1). When applying mixed cultures in MES, interactions between different organisms can be observed (Marshall et al. 2017).

#### Syntrophic interaction of anaerobic consortia

The term “syntrophy” dates back to the mid-twentieth century and was used to describe microbial cross-feeding (Fildes 1956). Today, syntrophy describes the cooperative growth of two or more species which can only utilize a substrate in mixed cultures, but not as a single species in pure culture (Stams et al. 2006). A generic association can be the supply of growth factors (e.g., vitamins) of one microorganism that enhances the growth of another microorganism in an exchange of substrates, or by the elimination of toxic products (Nozhevnikova et al. 2020), enabling the growth of both species. However, the syntrophic consortium in anaerobic environments is better described by the interdependence of metabolic pathways of species in their utilization of the available free energy. Initially, the organic degradation starts with hydrolytic and fermentative bacteria, breaking down polymeric substances, such as polysaccharides, proteins, and lipids, into mono- and disaccharides, amino acids, fatty acids, and alcohols. The intermediates are metabolized further by proton-reducing acetogenic bacteria into hydrogen (H_2_), carbon dioxide (CO_2_), formate and acetate (Nozhevnikova et al. 2020). In environments, where nitrate, sulfate, and iron are readily available, methanogens are usually outcompeted by nitrate, sulfate, or iron reducers, as these terminal electron acceptors allow a larger energy yield than methanogenesis (Morris et al. 2013). However, if these respirable substances are exhausted, CO_2_ constitutes the only terminal electron acceptor to oxidize electron carriers. Then, the methanogens and acetogens convert the end products of organic degradation into methane and acetate (Drake et al. 2002, Rosenberg et al. 2012).

For the total anaerobic decomposition of organic matter, hydrogen, as the end product, must remain at a low partial pressure (< 10 Pa) for a thermodynamically feasible degradation, as it allows the formation of H_2_ from nicotinamide adenine dinucleotide hydrogen (NADH), leading to a higher ATP gain for the primary fermentative bacteria (McInerney et al. 2009, Johnravindar et al. 2021). In the presence of a higher H_2_ concentration, the Gibbs free energy of the exergonic reactions for ATP-synthesis turns positive, leading to an accumulation of fatty acids. This results in an acidic pH shift, causing an inhibition of methanogens, and finally a termination of the organic degradation (Schink 1997).

#### Electron transfer mechanism

For the syntrophic interaction of microorganisms, the available energy is transferred by an electron movement across membranes, or by extracellular electron carriers. Three interspecies electron transfer (IET) mechanisms can be distinguished: (1) the indirect electron transfer (IDET) uses soluble or gaseous substances to transfer electrons between microorganisms, (2) the direct interspecies electron transfer (DIET) mechanism requires a physical contact between two species for the electron transfer, and (3) the mediated electron transfer (MET) utilizes electron carrying mediators that diffuse from mediator-producing to mediator-consuming cells to transport electrons (Park et al. 2018). In natural habitats and anaerobic digesters, the IDET via hydrogen and formate is the dominant IET mechanism between syntrophic organisms (Baek et al. 2018).

Both IDET and MET depend on the diffusion of the electron carrier from the donor to the acceptor cell. This limits the transport of electrons by the diffusion rates of, e.g., H_2_, formate, or another mediator. Through the modeling of Geobacter-mediated ethanol oxidation, with sulfate as an external electron acceptor, a metabolic advantage has been suggested for DIET over MET (Nagarajan et al. 2013). In contrast, another model considering electrochemical losses unique to DIET, such as activation losses of membrane-bound electron carriers (redox complexes), or the electrical resistance of nanowire or pili, lead to the conclusion that the IDET with formate as an electron carrier is thermodynamically more feasible than the DIET or the IDET with H_2_ (Storck et al. 2016). Depending on the application of the microorganisms, one IET might be more advantageous than the other.

## Comparison of pure and mixed culture MES performances

### Acetate as main product in MES

The combination of acetogens with a MES could offer a sustainable process for the production of platform chemicals such as acetate and further interesting products. In this context, the research approaches of the last years for the bio-catalyzed reduction of CO_2_ to acetate in MES differ with respect to the use of pure or mixed cultures as biocatalysts (Deutzmann and Spormann 2017; Wang et al. 2020; Yang et al. 2021; Roy et al. 2021). Table 1 presented in this section gives an overview of different acetate production rates related to the electrode surface (APR), the space-time yields (STY), and the operation conditions of several studies that used pure or mixed cultures in MES. Furthermore, studies with specific features and concepts will be examined and were added to the table for comparison.

**Table 1:** Overview of selected pure and mixed culture MES studies with a focus on acetate production parameters

Inoculation			Acetate production and efficiency			Operation and reactor design						Ref.
Culture type	(Dominant) organism(s)	Origin	APR [g∙day^−1^∙m^−2^]	STY [g∙day^−1^∙m^−3^]	CE [%]	Potential [V]	Operating time [days]	Mode and type	Volume [mL]	Cathode material	CS [cm^2^]	
Pure	*Clostridium ljungdahlii*	Engineered	17^b^	170	81.7	− 1.05 (Ag/AgCl)	7	Batch DC	250	Nickel-phosphide Carbon felt	25^b,d^	(Wang et al. 2020)
Pure	*Clostridium ljungdahlii*	DSM 13528	7.51	3.22^b^	39.15	− 0.9 (Ag/AgCl)	7^c^	Batch DC	200	Graphite felt + SS mesh	30^b^	(Bajracharya et al. 2015)
Pure	*Sporomusa ovata*	DSM 2662	15.79 ± 6.79^b^	176.89 ± 76^b^	83 ± 3	− 0.69 (SHE)	9^c^	Batch DC	250	Carbon cloth	28	(Chen et al. 2016)
Pure	*Moorella thermoautotrophica*	DSM 7417	3.50 ± 0.09^b^	36.4 ± 0.95^b^	65 ± 16	− 0.4 (SHE)	12	Batch DC	120	Carbon cloth	12.5	(Yu et al. 2017)
Pure	*Sporomusa ovata*	DSM 2662	47.3 ± 5.1	310 ± 33	62.7 ± 15	− 0.8 (Ag/AgCl)	10	Batch DC	250	Carbon cloth	16.25	(Krige et al. 2021)
Pure	*Sporomusa ovata*	DSM 2662	1.85 ± 0.13	21.46 ± 1.51^b^	83 ± 8	− 0.6 (Ag/AgCl)	7.5	Batch DC	125	Nickel Hollowfiber	14.5	(Bian et al. 2018)
Pure	*Clostridium ljungdahlii*	DSM 13528	34.6 ± 1.1	138 ± 4	42 ± 14	− 1.2 (Ag/AgCl)	8	Batch DC	250	Graphite rod	10^f^	(Roy et al. 2021)
Pure	*Desulfobacterium autotrophicum* HRM2	ATCC 43914	N/A	3.1^b^	83 ± 6	− 0.5 (SHE)	21^c^	Batch DC	120	Graphite rod	N/A	(Li et al. 2018)
Pure	*Moorella thermoacetica*	ATCC 39073	0.14 ± 0.006^b^	1.66 ± 0.08^b^	73 ± 6	− 0.5 (Ag/AgCl)	10	Batch DC	300	Graphite rod	35.5	(Faraghiparapari and Zengler 2017)
Pure	*Moorella thermoautotrophica*	DSM 7417	0.19 ± 0.016^b^	2.21 ± 0.19^b^	69 ± 6	− 0.5 (Ag/AgCl)	10	Batch DC	300	Graphite rod	35.5	(Faraghiparapari and Zengler 2017)
Pure	*Sporomusa ovata*	DSM 2662	0.46 ± 0.004^b^	5.44 ± 0.04^b^	81 ± 20	− 0.5 (Ag/AgCl)	10	Batch DC	300	Graphite rod	35.5	(Faraghiparapari and Zengler 2017)
Pure	*Sporomusa ovata*	DSM 2663	3.67 ± 1.09^b^	52.84 ± 15.65^b^	61 ± 13	− 0.69 (SHE)	14^c^	Batch DC	250	Graphite rod	36	(Aryal et al. 2017)
Pure	*Sporomusa acidovorans*	DSM 3132	2.65 ± 0.85^b^	38.14 ± 12.27^b^	69.9 ± 0.9	− 0.69 (SHE)	14^c^	Batch DC	250	Graphite rod	36	(Aryal et al. 2017)
Pure	*Sporomusa malonica*	DSM 5090	2.73 ± 0.29^b^	39.26 ± 4.24^b^	90.8 ± 14	− 0.69 (SHE)	14^c^	Batch DC	250	Graphite rod	36	(Aryal et al. 2017)
Pure	*Sporomusa ovata*	DSM 2662	13.75 ± 2.6^a,b^	270 ± 52^a^	66 ± 12	− 1.0 (Ag/AgCl)	34	Batch DC	250	Graphite rod + titanium mesh	49.5^f^	(Bajracharya et al. 2022)
Pure	*Sporomusa ovata*	DSM 2662	15.16 ± 1.42^b^	122.82 ± 11.5^b^	78.6 ± 5.6	− 0.69 (SHE)	14	Batch DC	250	PEDOT carbon cloth	20.25	(Aryal et al. 2018)
Mixed	N/A	Wastewater sludge	37.89	75.78^b^	41.25	− 1.1 (Ag/AgCl)	8^c^	Fed-batch DC	500	Graphite felt + Ss mesh	10	(Bajracharya et al. 2015)
Mixed	*Acetobacterium, Pseudomonas, Desulfovibrio, Sulfurospirillum*	Wastewater plant	66 ± 5.7	260 ± 30	84 ± 13	− 1.0 (Ag/AgCl)	10	Batch DC	250	Graphite plate	10^f^	(Roy et al. 2021)
Mixed	N/A	Wastewater plant	77.34^b^	618.75^b^	70^c^	− 1.1 (Ag/AgCl)	16	Batch DC	250	Carbon felt	20^b,d^	(Li et al. 2020c)
Mixed	Enriched with chemolithoautotrophs	Anaerobic sludge	622.5^e^	1634.06^b,e^	60 ± 0.2	− 0.8 (Ag/AgCl)	30	Fed-batch DC	200	Cobalt-oxide carbon felt	5.25^b,d^	(Anwer et al. 2021b)
Mixed	Enriched acetogenic culture	Anaerobic sludge	19 ± 1.7^a^	60 ± 6^a^	58 ± 5	− 1.26 (SHE)	40	Fed-batch DC	125	Carbon felt	N/A	(Patil et al. 2015)
Mixed	N/A	Anaerobic sludge	21.60 ± 1.87	N/A	68.8 ± 3.3	− 0.9 (Ag/AgCl)	N/A	Batch DC	25	Graphite felt	N/A	(Das et al. 2018)
Mixed	Enriched acetogenic culture	Anaerobic sludge	21^e^	1060^e^	63	− 1.2 ± 0.06 (Ag/AgCl)	172^c^	Conti. DC	200	Carbon felt	100^f^	(Arends et al. 2017)
Mixed	N/A	Anaerobic sludge	197.5 ± 10	2900 ± 500	55.5 ± 2.1	− 0.85 (SHE)	6.25^c^	Batch DC	100	Carbon paper + methylviologen	7^b,d^	(Anwer et al. 2021a)
Mixed	*Burkholderiales*, *Clostridiales*, *Natranaerobiales*	SPS + waste-water sludge	685 ± 30	372.64 ± 16.3^b^	100 ± 4	− 0.85 (SHE)	63	Fed-batch DC	250	RVC	1.36^f^	(Jourdin et al. 2015)
Mixed	Enriched acetogenic culture	Anaerobic sludge	0.56 ± 0.03^b^	25.3 ± 1.5^b^	45.3 ± 0.4	− 0.9 (Ag/AgCl)	20^c^	Batch DC	500	Graphite felt	224.25^b,d^	(Yang et al. 2021)
Mixed	*Sporomusa*, *Clostridium*, *Desulfovibrio*	Wastewater sludge	3.11^a,b^	121.6^a,b^	78.5	− 1.0 (Ag/AgCl)	7	Batch DC	450	Cylindrical graphite felt	176^d^	(Mateos et al. 2019)
Mixed	*Acetobacterium*, *Pseudomonas*, *Desulfovibrio*, *Sulfurospirillum*	Wastewater sludge	28^b^	250	60	N/A	14	Batch DC	280	Carbon felt	25^b,d^	(Song et al. 2019b)
Mixed	*Desulfopila corrodens* IS4, *Acetobacterium woodii*	DSM 15630, DSM 1030	10.67^b,e^	94.8^b,e^	90	− 0.5 (SHE)	20.8^c^	Batch DC	90	Graphite rod	8	(Deutzmann and Spormann 2017)
Mixed	*Clostridium ljungdahlii*, enriched acetogenic culture	DSM 13528, anaerobic sludge	23.81^b,e^	400^b,e^	53	− 1.0 (Ag/AgCl)	2	Batch DC	400	Graphite felt and rod	67.2	(Bajracharya et al. 2017)
Mixed	Mix of *Acetobacterium sp.*	Brewery wastewater	196.8^b,e^	18,720^b,e^	35	− 1.1 (SHE)	36	Conti. DC	50	RVC	48^f^	(LaBelle and May 2017)

*APR* acetate production rate, *ATCC* American Type Culture Collection, *Conti.* continuous, *CE* coulombic efficiency, *CS* cathode surface, *DC* double chamber reactor, *DSM* German Collection of Microorganism and Cell Culture, *N/A* not available, *PEDOT* poly(3,4-ethylene dioxythiophene), *Ref.* reference, *RVC* reticulated vitreous carbon, *Ag/AgCl* silver chloride electrode, *STY* space-time-yield, *SS* stainless steel, *SHE* standard hydrogen electrode, *SPS* stormwater pond sediments

^a^Average value is given in the study

^b^Calculated according to values from the corresponding reference

^c^Extracted from a graphic or table of the corresponding reference

^d^Geometric surface area: outer active surface area of the cathode

^e^Maximal value is given in the study

^f^Projected surface area: projected 2D active area of the 3D cathode

Most of the literature dealing with the topic of CO_2_ reduction to acetate in MES used mixed microbiological cultures as inoculums for the cathode chamber. However, the first studies in this field were mostly performed with pure cultures (Nevin et al. 2010; Nevin et al. 2011). Typical microorganisms used were acetogenic species including the model-organisms *S. ovata*, *C. ljungdahlii* and *M. thermoacetica* (Nevin et al. 2010; Bajracharya et al. 2015; Faraghiparapari and Zengler 2017; Yu et al. 2017; Wang et al. 2020). Especially *S. ovata* and other *Sporomusa* strains were often studied and are some of the most efficient acetogens with the ability of driving MES. Already the first studies of Nevin et al. (2010) showed coulombic efficiencies (CEs) up to 86 ± 21 % for the products acetate and 2-oxobutyrate. In comparison, recent studies from Krige et al. (2021) showed similar CEs in experiments with *S. ovata* and at the same time some of the highest APR (47.3 ± 5.1 g day^−1^ m^−2^) ever measured in pure culture MES. The improvement was achieved by a dual cathode configuration, in which carbon cloth and a titanium mesh were used as a cathode. Additionally, a synthetic biofilm of *S. ovata* was printed on the carbon cloth cathode (Krige et al. 2021). Besides *S. ovata*, also other strains of the *Sporomusa* genus are able to produce acetate in larger amounts during MES. Investigated *S. acidovorans* and *S. malonica* showed APRs of 2.65 ± 0.85 and 2.73 ± 0.29 g day^−1^ m^−2^, which are comparable with the result of *S. ovata* in the same MES setup (Aryal et al. 2017). Further studies investigated different *Clostridium* strains, focusing on the production of organic chemicals from CO_2_ by MES (Liu et al. 2018b; Wang et al. 2020). One of the main products was acetate with a production rate of up to 34.6 ± 1.1 g day^−1^ m^−2^ (Roy et al. 2021). Overall, the literature suggests that the electron transfer in pure culture MES occurs via H_2_ (IDET) and direct electron transfer (DET), depending on the number of suspended cells or biofilm formation (Aryal et al. 2017; Liu et al. 2018b; Krige et al. 2021).

In contrast to pure cultures, the MES with a mixed microbiological culture is primarily based on biofilm formation on the cathode. Due to the biofilm formation, the direct contact with the electrode surface is improved, which is reflected in an increased DET. Furthermore, mixed culture MES can benefit from synergistic effects such as positive effects of secreted secondary metabolites and improved production rates (Wael and An-Ping 2014; ter Heijne et al. 2019). On the other hand, the biofilm formation in mixed culture MES can affect the overall CO_2_ availability of the system negatively. To counteract this effect, there were initial studies that attempted to increase the CO_2_ availability in MES by a continuous recirculation of the gas headspace through the catholyte. The experiments resulted in a 44 % improvement of the space-time yield performance, with an average STY of 121.6 g day^−1^ m^−3^ (Table 1) and a maximum STY of 261 g day^−1^ m^−3^ (Bajracharya et al. 2017; Mateos et al. 2019).

To better evaluate the performance of a bio-catalyzed reduction of CO_2_ to acetate in MES with mixed cultures, it is necessary to consider the culture and inoculum compositions. In this context, the literature shows that the typical inoculum is anaerobic digester sludge from wastewater treatment plants, which, in turn, comes from the surroundings of the research facility (Song et al. 2019b; Li et al. 2020c; Izadi et al. 2021b). In addition, samples from brewery wastewater, stormwater pond sediments, and defined cultures are also utilized as inoculum (Jourdin et al. 2015; Deutzmann and Spormann 2017; LaBelle and May 2017). Commonly, the undefined mixed cultures are selectively enriched to establish a stable performing community for the acetate production, and to avoid a competition of acetogenesis and methanogenesis in MES (Marshall et al., 2013). Patil et al. (2015) were able to completely stop the production of methane over 2 months by the used enrichment protocol, which also led to an average APR of 19 ± 1.7 g day^−1^ m^−2^. Compared with other unmodified carbon cathode and reactor concepts, this average APR is supported by a good coulombic efficiency value of 58 ± 5 % recovered in acetate (30 ± 10 in H_2_).

Besides the simple enrichment of acetogenic organisms, several studies also analyzed the exact composition of mixed cultures in MES by genomic analysis techniques. As a result, important families and genera were identified, and differences between the composition of an inoculum and the final sample were revealed. Among others, the core community, which is usually present in acetogenic MES, includes *Acetobacterium*, *Pseudomonas*, *Clostridium*, *Sporumusa*, *Desulfovibrio*, and *Sulfurospirillum* (Mateos et al. 2019; Song et al. 2019b; Roy et al. 2021; Izadi et al. 2021b). The genera *Sporumusa* and *Clostridium* have been identified as acetogenic bacteria with species that are confirmed as bioelectrochemically active. Thus, they most likely play an important role in the electron transfer and the acetate production of the system (Nevin et al. 2011; Tremblay and Zhang 2015; Engel et al. 2020). The genus *Pseudomonas* has been reported in several MES studies. It is assumed to be involved in the extracellular electron transfer process through its hydrogenase enzymes and the secretion of mediators (Mateos et al. 2019). *Desulfovibrio* are often described as the main sulfate-reducing organisms in the mixed culture community, which are enriched because of sulfate-containing media. However, a positive effect on MES is assumed, as members of this genus can accept electrons directly from an electrode to reduce H^+^ ions to H_2_. Furthermore, some *Desulfovibrio* strains are able to metabolize formate. Both products could be used by the acetogenic bacteria in the cathodic biofilm to further increase the acetate production (Labelle et al. 2020; Roy et al. 2021). Nevertheless, the different studies exhibit a large variation among the microbiological communities, due to the influence of different inoculum origins and operation modes of MES. Simultaneously, this fact indicates the probably untapped potential of mixed cultures for the production of acetate in MES.

One possible way to improve the comparability of the performance of mixed culture MES is to use defined mixed cultures as biocatalysts. This approach has been adopted in a few studies, and some of them only rely on the additional supplementation of an undefined mixed culture with selected strains. Thus, Bajracharya et al. (2017) supplemented a long-term operation of MES with a pre-culture of *C. ljungdahlii* to ensure the activity and presence of homoacetogens. With this semi-defined mixed culture, a maximum APR of 23.81 g day^−1^ m^−2^ was achieved within two days of a long-term experiment operated over 300 days. This contrasts with the experimental series of Deutzmann & Spormann (2017), who investigated the application of a defined co-culture in MES. For this purpose, they utilized *Desulfopila corrodens* IS4 for the production of the intermediate hydrogen by an electron uptake at the cathode. As a counterpart, the acetogenic bacterium *Acetobacterium woodii* was used for the acetate production since the genus *Acetobacterium* occurred in many mixed culture community analyses (Patil et al. 2015). Although the co-cultivation of these organisms resulted in an only low biofilm formation, since most of the electron transfer occurred via hydrogen, an APR of 10.67 g day^−1^ m^−2^ was achieved. This suggests that only a combination of a variety of specialized organisms leads to a functional biofilm for DET in MES.

Whether pure or mixed cultures perform better in MES is a difficult question to answer. It is best solved by comparing both conditions in an identical experimental setup. To date, to the best of our knowledge, there are no known publications directly comparing pure and mixed culture MES under same conditions. Common differences include pH value, reactor design, and media composition, which are adapted to the needs of the organisms (Bajracharya et al. 2015; Roy et al. 2021). However, if minor differences are overlooked, the results show a positive influence of the microbial mixed culture in MES. Experiments with CO_2_-rich brewery gas showed an improved production of acetate with an enriched mixed culture, dominated by the *Acetobacterium* species compared to a pure culture of *C. ljungdahlii*. The mixed culture with an APR of 66 ± 5.7 g day^−1^ m^−2^ outperformed the pure culture by nearly doubling the APR of 34.6 ± 1.1 g day^−1^ m^−2^. This was also reflected in a significant difference in electron recovery. In mixed culture MES, more than 84 ± 13% of the electrons were recovered in acetate, whereas the pure culture recovered only 42 ± 14% (Roy et al. 2021).

In many cases, the reactor design varies regarding the type of cathode used, as the goal of numerous studies is to find new cathode materials, to optimize known materials with different coatings, or to try new cathode setups for MES (Wang et al. 2020; Bajracharya et al. 2022). Mostly, the cathode optimization is performed, independent of the fact, whether mixed or pure cultures are used. Still, the general literature on MES tends to be dominated by mixed cultures, which is also reflected in the optimization experiments. In addition to the typical graphite rod or carbon cloth cathode, the initial experiments were conducted with an additional stainless-steel mesh, gas diffusion electrodes, or reticulated vitreous carbon (RVC) as the electrode (Bajracharya et al. 2015; LaBelle and May 2017; Fontmorin et al. 2021).With 685 ± 30 g day^−1^ m^−2^ Jourdin et al. (2015) were able to achieve one of the highest maximum APRs by utilizing an RVC electrode in mixed culture MES. When considering the result, the small projected surface area of 1.36 cm^2^ should be taken into account. Another study with modified electrode surfaces obtained a maximum APR of 622.5 g day^−1^ m^−2^ in mixed culture MES. Thereby, they used a combination of carbon felt and stainless-steel mesh, both coated with cobalt oxide (Anwer et al. 2021b). Nevertheless, it is difficult to compare experiments with different setups in terms of the influence of pure or mixed cultures in MES. Therefore, in addition to the acetate production, it is worthwhile to further investigate the methane production by methanogenesis to get a better insight into pure and mixed culture MES.

### Methane as main product in MES

Methane is the main compound of natural gas and can be efficiently utilized for heat and electricity production. In combination with methanogens, MES offers a sustainable and selective process for the production of methane, and could serve as storage technology for excess electricity (Enzmann et al. 2018; Gomez Vidales et al. 2019). In literature, the approach of the bio-catalyzed reduction of CO_2_ to methane is performed with both pure and mixed cultures. For this section, MES studies with the highest methane production rate (MPR) and space-time yield (STY) were selected and listed by the usage of pure or mixed cultures (Table 2). Studies with specific features and upstream concepts were added to the table for discussion and comparison.

**Table 2:** Overview of selected pure and mixed culture MES studies with a focus on methane production parameters

Inoculation			Methane production and efficiency			Operation and reactor design						Ref.
Culture type	(Dominant) organism(s)	Origin	MPR [mmol∙day^−1^∙m^−2^]	STY [mmol∙day^−1^∙m^−3^]	CE [%]	Potential [V]	Operating time [days]	Mode and type	Volume [mL]	Cathode material	CS [cm^2^]	
Pure	Methanococcus maripaludis	DSM 14266	8.81 ± 0.51	67 ± 4^b^	10 ± 0.03	− 0.7 (SHE)	3.0c	Batch DC	300	Graphite rod	22.8^b,d^	(Mayer et al. 2019)
Pure	Methanococcus maripaludis	DSM 14266	64.8 ± 8.1	3576 ± 447^b^	63.1	− 1.1 (Ag/AgCl)	18.75	Batch BC	1000	Carbon fibers in cylindrical rods	552^b,d^	(Enzmann et al. 2019a)
Pure	Methanosarcina barkeri	N/A	44.0e	4950e	N/A	− 1.2 (Ag/AgCl)	1.5	Batch DC	75 ml 2 ml cage	Cellulose ester membrane with dispensed CNT	2.25^b,d^	(Bai et al. 2020)
Pure	Methanococcus maripaludis	Engineered (Costa et al., 2010)	812.29^b^	90,163^b^	99.0	50 mA galv.	7.3–7.9^c^	Conti. DC	100	CA cathodes with a NiMo-alloy	111^d^	(Kracke et al. 2021b)
Pure	Methanococcus maripaludis	DSM 14266	10.24	230	113.6	− 1.1 (Ag/AgCl)	8.33	Batch BC	50,000	Cylindrical carbon layings	30,000^d^	(Enzmann and Holtmann 2019)
Pure	Methanothermobacter thermautrotrophicus	DSM 1053	43.6	1635^b^		− 1.5 V w/o reference	2.0	Batch SB	10	Carbon paper with carbon layer	3.0^d^	(Sato et al. 2013)
Pure	Methanococcus maripaludis	Engineered (Costa et al., 2010)	1131.6^b^	56,557^b^	> 90^a^	− 0.65 (SHE)	42.0	Conti. DC	100	NiMo coated graphite rod	50	(Kracke et al. 2020)
Mixed	Desulfopila corrodens IS4, Methanococcus. maripaludis	DSM 15630, engineered (Costa et al., 2010)	144–216	1092–1638^b^	N/A	− 0.5 (Ag/AgCl)	0.28c	Batch DC	105.5	Graphite rod	8	(Deutzmann and Spormann 2017)
Mixed	N/A	Enriched methanogenic culture	44	3168^b^	98.0	− 0.7 (SHE)	29.0	Batch DC	500	Graphite rod	36	(Vasiliadou et al. 2021)
Mixed	N/A	Anaerobic sludge	24.64^b^	33,260^b^	70^a,b^	− 0.9 (NHE)	22^b^	Conti. PMR	650	Graphite plate	8775(s)^b^	(Gomez Vidales et al. 2019)
Mixed	N/A	Anaerobic sludge	28.91b	39,029b	86a,b	− 0.7 (NHE)	14.0b	Conti. PMR	650	Graphite plate	8775(s)^b^	(Gomez Vidales et al. 2019)
Mixed	Methanobacterium Coriobacteriia, Desulfovibrio, Desulfomicrobium	Sewage treatment plant	281.60	1625^b^	89.4	− 0.7 (Ag/AgCl)	90.0	N/A DC	260	PEDOT on graphene oxid carbon cloth	15^b,d^	(Li et al. 2020b)
Mixed	N/A	Wastewater treatment plant	3661.88^b^	21,700	80.5	− 0.8 (SHE)	7.0	Conti. CSTR	135	Carbon felt	4^b,d^	(Nelabhotla and Dinamarca 2019)
Mixed	Methanobacterium, Methanobrevibacter Sporomusa, Petrimonas	Effluent from microbial electrolysis cell	5799.18^b^,^e^	58,197^b^,e	45.7	5 A galv.	8	Batch MBBR	4500	Stainless steel perforated mesh spiralized	450^b,d^	(Cai et al. 2022)
Mixed	Methanobacterium palustre, Methanobacterium aarhusense	Anaerobic sludge	168.03^b^	7860^b^	99.0	− 0.7 (NHE)	24	Semi-conti. FPR	620	Graphite felt	361^f^	(van Eerten-Jansen et al. 2013)
Mixed	Methanothrix, Azonexus	Anaerobic sludge	1069.65^b^	5375^b^	97.5	− 0.5 (SHE)	27	Conti. MEC	800	Graphite plate	40.2^f^	(Liu et al. 2020)
Mixed	Methanobacterium sp., Proteobacteria	Effluent from an anaerobic digester	15.35	11,310^b^	68.9 ± 0.8	− 0.8 (SHE)	80	Conti TCR	420	N/A	5700	(Batlle-Vilanova et al. 2015)
Mixed	N/A	Active sludge	6800.63^b^	40,300		− 0.65 (SHE)	2.0c	Conti. CSTR	135	Carbon felt	8	(Nelabhotla et al. 2020)
Mixed	Methanobacterium, Geobacter, Peptostreptococcaceae	Bog	110,655.74^b^	3,319,672^b^	93	− 0.7	7.0–14.0	Batch SC	5	Graphite plate	1.5^f^	(Siegert et al. 2014a)
Mixed	Methanobacterium, Methanobrevibacter, Geobacter, Sporumosa, Peptostreptococcaceae	Anaerobic digester sludge	81,967.21^b^	2,459,016^b^	114	− 0.7	7.0–14.0	Batch SC	5	Graphite plate	1.5^f^	(Siegert et al. 2014a)
Mixed	N/A	Anaerobic sludge	360.7^b^	28,852^b^	69.4	− 1.3 (Ag/AgCl)	3.0	Batch TCR	25	Graphite felt	20	(Liu et al. 2017)
Mixed	Methanobacterium, Methanocorposculum, Betaproteobacteria	Mix of anaerobic sludges	614.75^b^	40,984b	54.0^c^	10 A/cm2 galv.	34.0c	Batch PR	33	Granular activated carbon	64,940,000^b,f^	(Liu et al. 2018a)
Mixed	Methanobacterium, Methanocorposculum, Betaproteobacteria	Mix of anaerobic sludges	2663.93^b^	176,230^b^	66.0^c^	35 A/cm2 galv.	19.0^c^	Batch PR	33	Granular activated carbon	64,176,000^b,f^	(Liu et al. 2018a)
Mixed	N/A	Anaerobic sludge	2633.6b	9754b	52.3 ± 3.1	− 0.8 (Ag/AgCl)	7	Batch SC	270	Magnetite/zeolite carbon cloth	10f	(Vu et al. 2020)
Mixed	Methanobrevibacter arboriphilus	N/A	103.2 ± 4.94	3550 ± 170	69.0	− 0.8 (SHE)	7	Batch DC	250	Carbon felt pieces	86d	(Dykstra and Pavlostathis 2021)
Mixed	Methanobacterium, Methanobacterium palustre	Anaerobic sludge	384.3	334,341^b^	64.4	− 0.7 (SHE)	7.16	Batch D^c^	200	Graphite felt	4d 1740 (s)	(Baek et al. 2017)
Mixed	Methanothermobacter wolfeii	Anaerobic sludge	380.0	3078^b^	96.85	− 0.85 (Ag/AgCl)	137	Batch MR	1000	Carbon disk	81.01^d^	(Song et al. 2019a)

*BC* bubble column, *CA* carbon aerogel, *CNT* carbon nanotubes, *CS* cathode surface, *Conti.* continuous, *CSTR* continuous stir tank reactor, *CE* coulombic efficiency, *DC* double-chambered reactor, *DSM* German Collection of Microorganism and Cell Culture, *FPR* flat plate electrochemical reactor, *galv.* galvanostatic, *MBBR* electro-moving bed biofilm reactor, *MEC* continuous-flow microbial electrolysis cells, *MPR* methane production rate, *MR* membraneless reactor, *NiMo* nickel-molybdenum, *N/A* not available, *PEDOT* poly(3,4-ethylene dioxythiophene), *PMR* Plexiglas membraneless reactor, *PR* plate reactor, *SHE* standard hydrogen electrode, *NHE* normal hydrogen electrode, *TC* triple chambered reactor, *TCR* three-chambered reactor, *SB* serum bottle, *Ag/AgCl* silver chloride electrode, *SC* single-chambered reactor, *STY* space-time-yield, *w/o* without

^a^Average value is given in the study

bCalculated according to values from the corresponding reference

^c^Extracted from a graphic of the corresponding reference

^d^Geometric surface area: total outer active surface area of the cathode

^e^Maximal value is given in the study

^f^Projected surface area: projected 2D active area of the 3D cathode

From our literature research, the first difference between pure and mixed cultures is a considerably higher number of publications on methane-producing mixed cultures than on pure cultures, as reflected in Table 2. In contrast in biotechnology, mixed cultures have been gradually replaced by pure cultures, as the process control of the cultivation is simplified by the exclusion of interspecies interaction. In applying pure cultures, one cultivation optimum can be established, while a contamination of the fermentation product is avoided. However, it is estimated that 90–99.8% of microbes cannot be grown in a pure culture with the current technology, hence a great number of microorganisms cannot be exploited for biotechnology as a pure culture (Streit et al. 2004). Additionally, natural mixed cultures can have several advantages over pure cultures, as they are (i) more flexible and resilient, (ii) can utilize a broad spectrum of low-cost substrates, and (iii) generate possible higher production rates through synergistic effects (Wael and An-Ping 2014; Zhao et al. 2015; Jiang et al. 2017; ter Heijne et al. 2019).

Another characteristic of mixed cultures is the ability to form biofilms on biocompatible surfaces. Frequently, studies related to a pre-inoculated cathode in a mixed culture suspension until a biofilm was established (Baek et al. 2017; Schlager et al. 2017; Nelabhotla and Dinamarca 2019). The positive influence of a biofilm on the performance of MES was demonstrated by repeatedly inoculating a cathode for developing a biofilm, resulting in higher MPR each cycle (Baek et al. 2017; Vasiliadou et al. 2021). The benefit of an established biofilm is a cell-to-electrode contact, enabling a DET. The unmediated integration of electrons into the metabolism of microorganisms has the advantage of omitting the electrochemical production of hydrogen as an intermediate for methanogenesis. Although H_2_ can be efficiently utilized by hydrogenotrophic methanogens as an energy carrier, it has many disadvantages as a gas in biotechnological processes, such as (i) the temperature-dependent H_2_ solubility in a culture medium, (ii) the loss of H_2_ through the fuming of bubbles, and (iii) an overall higher energy input for the electrochemical H^+^ reduction than in a direct electron transfer system (Sonne-Hansen et al. 1999; Siegert et al. 2014b; Kracke et al. 2021a). A drawback of biofilms is the slow development, which can lie between weeks to several months (Cheng et al. 2009; Liu et al. 2017), hence a slow start-up of MES is observed (Jiang and Zeng 2019). Another critical aspect is the obtained thickness of the biofilm, which determines the maximal current density (Jourdin et al. 2015; Claassens et al. 2019). By reversing the applied potential, Li et al. (2019) were able to reduce the startup time by 40% and achieve a higher current density, which suggested a thicker biofilm. Initially, the electrode was developed as a bioanode, with acetate as an electron donor promoting an anodic biofilm formation. In the cathodic mode, the bioelectrode showed an improved cathodic biofilm formation, attributed to the symbiotic association of an anodic and a cathodic consortium. Compared to the control, the reverse biocathode produced 1.2 times higher amounts of methane. In contrast, almost all pure methanogen cultures are suspension cultures in which the IDET is via H_2_.

To target the question, whether pure or mixed cultures perform better in MES, a comparison of both conditions in the same system would be necessary. As of today, our literature research has not revealed a study of that kind for methane-producing MES systems. However, one possibility is the comparison of production rates and yields of experiments with either pure or mixed cultures, but with similar parameters. Vasiliadou et al. (2021) and Mayer et al. (2019) used both similar systems in comparable operational modes. Herein, the enriched mixed culture produced 3.5-fold more methane than *Methanococcus maripaludis* S2 (considering the difference in active cathodic surface). For the pure culture, the CE was substantially lower compared to the mixed culture. Other species investigated by Mayer et al. reached lower production rates, but higher CEs. These results indicate that in a standard type reactor (H-cell reactor) with unmodified cathodes, mixed cultures outperform pure cultures regarding methane production rate and CE.

However, simply comparing mixed and pure cultures with similar parameters can be misleading. One system alone might not be optimal for both conditions, as the prevailing electron transfer differs between each condition, assuming that pure cultures are suspended rather than sessile cells (Beese-Vasbender et al. 2015). The group of Bai et al. (2020) aimed to promote a direct cell-to-cathode contact for a pure culture, as the theoretical thermodynamic energy input of DET is lower than that for IDET via H_2_ (van Eerten-Jansen 2014). For the DET, a cage-type electrode was constructed, which facilitated the attachment of *Methanosarcina barkeri* and simultaneously enabled the nutrient supply. The results showed that DET was the main route for methanogenesis when potentials were higher than − 0.6 V (vs. Ag/AgCl). At − 0.6 V or lower, the proportion of IDET dominated the methane production. Noticeably, the MPR increased more than 10-fold as the potential was increased from − 0.4 to − 1.2 V (Ag/AgCl), showing that the methane synthesis of *M. barkeri* is more effective via a H_2_ electron transfer mechanism than via DET. Overall, the performance of the cage cathode enabled MPR comparable to other studies, but at higher potentials, which implies the requirement of a higher energy input.

Instead of promoting a DET in pure cultures, an enhanced electrochemical H_2_ production is another approach for improving production rates in MES, as hydrogenotrophic methanogens are efficient H_2_ scavengers (Tartakovsky et al. 2011; Jourdin et al. 2016; Kracke et al. 2021b; Bajracharya et al. 2022). Kracke et al. (2019) successfully demonstrated that inexpensive metal alloy catalyzes the H^+^ reduction at low overpotentials with a 100% selectivity for H_2_. In a subsequent study, the application of a NiMo graphite rod led to one of the highest STY reached for pure culture, with 56,557 mmol∙day^−1^∙m^−3^ up to date (Table 2) (Kracke et al. 2020). Furthermore, the group focused on the geometrical optimization of the cathode. Cylindrically shaped carbon aerogel (CA) cathodes with a NiMo-alloy, but varying amounts of cavities, resulted in different surface areas and revealed the influence of the current density on methanogenesis in MES (Kracke et al. 2021b). A stable methane production was reached at a low current density within 24 h, whereas high current densities led to a fluctuating production. The proportion of unused hydrogen also increased with an elevated current density. Both findings were explained by the passivation of the electrode surface through H_2_ bubbles, a low pH at the electrode surface, and a loss of hydrogen through bubble formation at higher hydrogen evolution reaction (HER) rates (Angulo et al. 2020). At lower current potentials, the consumption rate of hydrogenotrophs is above the HER rate, resulting in a rapid H_2_ consumption and in an avoidance of bubble development. The STY of 90,160 mmol∙day^−1^∙m^−3^ of the study was the highest among pure cultures, and one of the highest compared to mixed culture conditions, concluding that the local physical conditions at the electrode limit the metabolic capacity of methanogens rather than their metabolic capacity.

Cathode optimization has also been performed under mixed culture conditions. The aim of the studies was either to find new cathode material, or tuning known material with metals or mediators (Alqahtani et al., 2018; Vu et al., 2020; Yang et al., 2020). With regard to stable long-term performance, Liu et al. (2018a) examined granular activated carbon as a cathode material, which yielded high MPR and STY. With 66%, the CE was one-third lower than for the previously described pure culture. Hydrogen was not detected, which supports the DET mechanism or efficient H_2_ scavenging, but also the occurrence of side reactions channeling the electron flow towards an undesired product, which reduces the efficiency of methane production as expressed in the lower CE (Yang et al. 2020). A mix of products was observed in other studies as besides methanogens (Song et al. 2019a). A diverse group of microorganisms were present in native mixed cultures that were capable of reducing CO_2_ to acetate or higher volatile acids in MES reactors (Arends et al. 2017; Batlle-Vilanova et al. 2017; Gavilanes et al. 2019; Vassilev et al. 2019; Mateos et al. 2019). To avoid more than one product, the consortium of microorganisms must be selected beforehand. As methane-producing MES are dominated by hydrogenotrophic methanogen species (ter Heijne et al. 2019), the selection can be realized by incubating the mixed culture in defined media, with H_2_ as the sole energy source. Additionally, the process parameters have to be adjusted to promote methane formation over other products. The applied potential during startup determined the electron transfer mechanisms of the biocathode with lower initial potentials (− 0.7, − 0.8 V vs Ag/AgCl) enabling DET, whereas higher potentials (− 0.9, − 1.0, − 1.1 V vs Ag/AgCl) promoted IDET (Li et al. 2020a). For the methane production, potentials higher than − 0.95 V (vs. Ag/AgCl) should be applied to avoid by-products such as acetate (Jiang et al. 2013). Furthermore, a stable pH at slight acidic to neutral provides the optimal condition for methanogenesis (Visser et al. 1993).

By comparing the mode and the type of reactor between pure and mixed cultures, one noticeable difference is the application of various reactor types for mixed cultures, ranging from single-chambered (SC) to classical double-chambered (DC) reactors. Also, three-chambered reactors (TC), plate reactors, CSTR, and membraneless reactors have been applied (Batlle-Vilanova et al. 2015; Liu et al. 2017; Gomez Vidales et al. 2020). The diversity of reactor types is generally attributable to the greater amount of research with mixed cultures, but is also driven by the idea of integrating MES into existing wastewater treatment plants and anaerobic digesters (AD) (He et al. 2019; Vu and Min 2019). A study by Nelabhotla and Dinamarca (2019) showed that using reject wastewater from anaerobic digesters increases the methane content of biogas > 90%. Additionally, the influence of the hydraulic retention time (HRT) was investigated for an integrated AD-MES process (Nelabhotla et al. 2020). The highest production rate was detected at 3- and 6-h HRT, although, correlated to the feed, 18-h HRT had the optimal MPR and the highest COD removal. The study outlined that besides high MPR, other values have to be considered when integrating MES into waste streams. As another reactor type, a continuous stir tank reactor (CSTR) was utilized frequently, but with the limitation in the gas distribution for upscaling (Rittmann et al. 2012; Kim et al. 2013). The optimal reactor type for gas fermentation is the bubble-column reactor and the fixed bed reactor with an increased gas retention time and no requirement for any additional mixing (Lee et al. 2012; Alitalo et al. 2015; Kougias et al. 2017). The developed electrolytic-hydrogen-fed moving bed biofilm reactor (electro-MBBR) by Cai et al. (2022) combined an electrochemical cell with an MBBR on top and was designed to increase the hydrogen mass transfer for an efficient methane production. The achieved maximal MPR was one of the highest, but the CE for methane was below 50%.

Furthermore, the characteristics of mixed consortia contribute to the diversity of applicable reactor types, as mixed cultures are more oxygen-tolerant due to facultative anaerobic bacteria than pure cultures (Li et al. 2019). This feature allows the utilization of SC reactors and membraneless reactors, which have the advantage of a lower internal resistance, resulting in a decrease of the current drain, an unrestricted ion transport, and lower material costs by omitting a cation exchange membrane (Gomez Vidales et al. 2019; Song et al. 2019a). The highest MPR and STY listed were reported from SC reactors, but due to the small volume of 5 mL (Siegert et al. 2014a), the electrode surface to reactor volume ratio is large by comparison, resulting in high MPR and STY.

For pure cultures, the dominant reactor type is a double-chambered reactor. An exception is the reactor developed by Enzmann et al. (2019b), which consists of a bubble column as the cathodic chamber and a surrounding basin as the anodic chamber. Its design enables a flexible use of electrode and membrane material, while it is scalable for industrial production through dimensionless numbers. The type of reactor is advantageous for gaseous substrates and can be applied as a microbial fuel cell (MFC) or MES. In a subsequent study, the long-term stability of the system was demonstrated as well as its recovery after failure scenarios, such as a potential or a gas shut-off. Furthermore, a scale-up of the reactor was performed, which showed high amounts of methane, but comparably low MPR and STY.

The bubble column reactor was operated in a batch mode, which is the predominant mode for a pure culture MES. Differently, both studies of Kracke et al. (2020 and 2021b) used a continuous mode, which might be another reason for the high MPR and STY. For mixed cultures, the effect of the operational mode was tested. Switching the operational mode from batch to continuous increased the methane production rate and the STY 3-fold (Batlle-Vilanova et al. 2015). A possible explanation is a steady pH throughout the experiment, which is beneficial for the organism’s metabolism (Jones et al. 1983; Izadi et al. 2021a).

The microbial composition of the original mixed cultures changes when used in BES, as a genomic analysis showed (Yang et al. 2020). Especially for the group of archaea, the hydrogenotrophic *Methanobacterium* genus was primarily identified in mesophilic mixed cultures (van Eerten-Jansen et al. 2013; Batlle-Vilanova et al. 2015; Alqahtani et al. 2018; Li et al. 2020b). Further, the genus *Methanobrevibacter* and the order *Methanosarcinales* were ascertained, but at lower percentages (Jiang et al. 2014; Liu et al. 2020; Yang et al. 2020; Dykstra and Pavlostathis 2021). The group of bacteria was more diverse in MES cultures (van Eerten-Jansen et al. 2013; Baek et al. 2017). A readily occurring phylum is the *Proteobacteria*, which is one of the largest phyla in the domain bacteria (Batlle-Vilanova et al. 2015; Yang et al. 2020). The *Proteobacteria* are subdivided into many more classes, orders, and genera, but none can be specifically determined for being dominant in the cathodic chambers of MES, except for the genus *Geobacter* and *Sporumosa* (Siegert et al. 2014a; Li et al. 2019; Cai et al. 2022). Furthermore, *Bacteroidetes* and *Firmicutes* have been proposed to produce H_2_ in autotrophic electroactive biofilms (Xafenias and Mapelli 2014; Wang et al. 2021). Another frequently identified bacteria genus was *Desulfovibrio,* which could be involved in hydrogen production at potentials smaller than − 0.44 V (vs. NHE) (van Eerten-Jansen et al. 2013; Li et al. 2020b). However, not all organisms could be identified, and the role of each microorganism can be different in mixed cultures and in pure cultures, leaving a huge untapped potential for improving MPR by defined mixed cultures in the future.

## Conclusion and outlook

Over years of intensive global efforts, MES has been developed more and more to be turned into a potential replacement for specific branches of the chemical and energy industry based on fossil resources. Especially the potential of microbial electrosynthesis to use CO_2_ from industrial exhaust gases for production holds many possibilities and would be a further step towards bioeconomy. However, a detailed understanding of the advantages and disadvantages of pure and mixed cultures in MES is essential for industrial applications, but until today, it still remains incomplete. Research activities of recent years show a quantitative focus on mixed culture MES compared to pure culture MES. In a standard bioelectrochemical H-cell reactor, pure cultures are outcompeted by mixed cultures regarding production yields and the efficiencies of acetate and methane. Various reactor types were used with mixed culture conditions, enabling a broad spectrum of applications. Furthermore, the common use of undefined mixed cultures shows low risks in terms of contamination hazards, but possibly variations in the composition of cultures over the cultivation time. This is also reflected in the production specificity of undefined mixed cultures, which is lower compared to pure culture MES, creating an obstacle for an industrial use of undefined mixed cultures for a single target production. In contrast, the low susceptibility to contamination could be exploited to use MES with undefined mixed cultures as an integral downstream process of production plants with CO_2_ exhaust gases or wastewater treatment plants. For pure cultures, high production rates and space-time yields were achieved in a well-adjusted H-cell reactor, demonstrating the competitiveness of pure conditions in MES systems. Due to a better product specificity, pure cultures could be more suitable for industrial applications than undefined mixed cultures. However, first experiments in a scale-up reactor for a methane production showed a significantly lower production compared to mixed cultures, revealing the necessity of a reactor optimization for a scale-up production. However, the scale-up that is highly important in order to realize the potential discussed above in terms of decarbonization and electrification.

Based on the conducted literature research and own experience, one of the key challenges for the future of MES could be the development of a perfectly adjusted defined mixed culture to overcome some of the main problems of MES. Therefore, further studies need to focus on the role of different species in a consortium used for MES and on the impact of interspecies interactions between members of the consortium. The knowledge gained through these studies could be used to finally establish a defined mixed culture for an industrial use of MES.
